# A Tale of Two “Citis”: Appendicitis Versus Crohn’s Appendicitis

**DOI:** 10.7759/cureus.62803

**Published:** 2024-06-20

**Authors:** Bhavan Peddi, Rajiv Raj, Manoj Prabhakar, Ramya Ramakrishnan

**Affiliations:** 1 General Surgery, Sri Ramachandra Institute of Higher Education and Research, Chennai, IND; 2 Surgery, Apollo Institute of Medical Sciences and Research, Chittoor, IND

**Keywords:** steroids, conservative management, skip lesions, acute appendicitis, crohn’s disease

## Abstract

Appendicitis is one of the most common emergencies worldwide. One of the rare causes of acute appendicitis is Crohn’s disease (CD). Management of appendicitis should not be decided in haste without a complete workup of the patient, including coexisting symptoms and past history. The appendix is essential for intestinal homeostasis, preventing the development of certain pathologies. It is important to correlate clinical and radiographic findings in diagnosing and managing Crohn’s appendicitis. The mainstay of management of CD with appendicitis involves the use of systemic steroids.

## Introduction

Appendicitis is one of the most common emergencies globally [1]. One of the rare causes of acute appendicitis is Crohn’s disease (CD) [2]. Appendicectomy has been the go-to modality whenever appendicitis has been diagnosed clinically or radiologically [3]. Conservative management is seldom tried by clinicians after diagnosis of appendicitis has been made since in-depth workup into identifying the cause and careful evaluation of other symptoms is lacking [4]. CD is a type of inflammatory bowel disease (IBD) that can affect any part of the gastrointestinal tract from mouth to intestine. It has many extraintestinal manifestations involving skin, joints, liver, kidney, eyes, etc. Crohn’s appendicitis or granulomatous appendicitis is one of the rarer presentations of IBD and is usually detected on a histopathology specimen after appendectomy [5]. Incidence is 0.128%-0.4% [6]. Here, we report a case of Crohn’s appendicitis in a 40-year-old female with a history of seronegative arthritis.

## Case presentation

**Figure 1:**
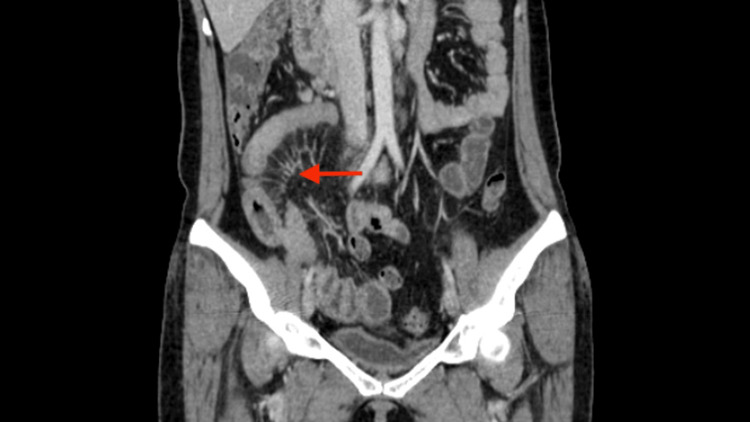
Mild mesenteric fat stranding suggestive of CD (red arrow) CD: Crohn's disease

A 40-year-old female with a history of seronegative arthritis (not on medication at present) presented with abdominal pain and vomiting for 20 days. The patient had bilateral lower limb pain and swelling for 20 days. The patient had a skin rash (reddish) on the bilateral lower and upper limbs for seven days. The patient also had a history of weight loss of 6 kg in one month. The patient has no history of fever, cough, cold, and bowel and bladder disturbances. The patient has a history of cerebrovascular accident (not on medication at present) and hypothyroidism (on Tab. Thyronorm 100 mcg once daily). On palpation, the patient had right iliac fossa (RIF) tenderness. The total counts are 10,000, CRP is 3.9, and ESR is 41. An ultrasonogram of the abdomen showed features of ileitis and mild ascites, and the appendix was not visualized separately. Contrast-enhanced computed tomography (CECT) of the abdomen showed thickened and edematous distal ileal loops and ileocecal junction with prominent adjacent vasa recta and mild mesenteric fat stranding (Figure 1).

**Figure 2:**
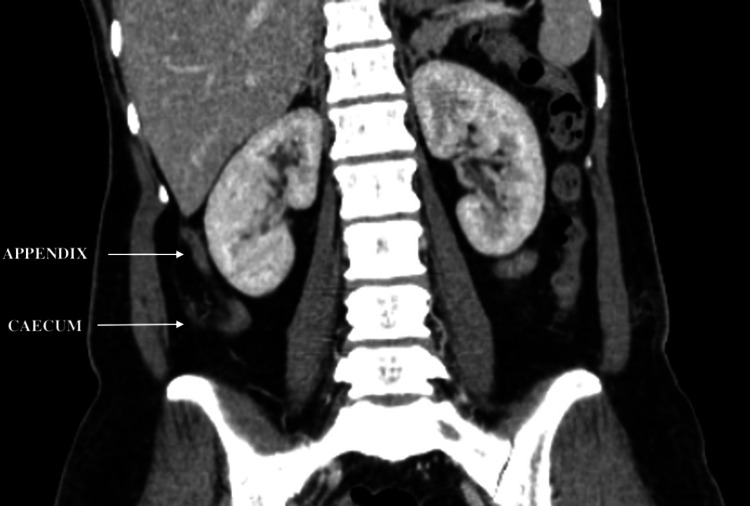
Inflamed subhepatic appendix arising from cecum suggestive of appendicitis (white arrows)

CECT of the abdomen also showed a few enlarged lymph nodes in the RIF and an inflamed subhepatic appendix (Figure 2).

**Figure 3:**
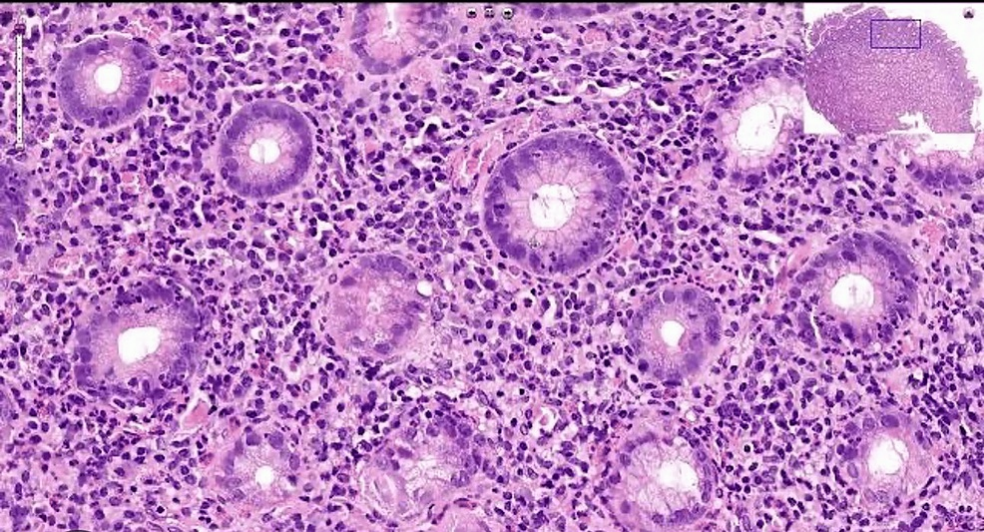
Gastric mucosa with neutrophils present on the gastric glands suggestive of chronic active gastritis

All these features suggested IBD-CD with Crohn’s appendicitis. Given CECT findings, it was decided to defer surgery and go ahead for further evaluation. Rheumatoid arthritis factor, anticitrullinated protein antibody, antinuclear antibody, antineutrophil cytoplasmic antibody, antiphospholipid antibody, and cryoglobulin were all negative. Upper GI endoscopy showed erosive gastritis with bulbar duodenitis. Biopsy showed severe chronic active gastritis with moderate intestinal metaplasia (Figure 3).

**Figure 4:**
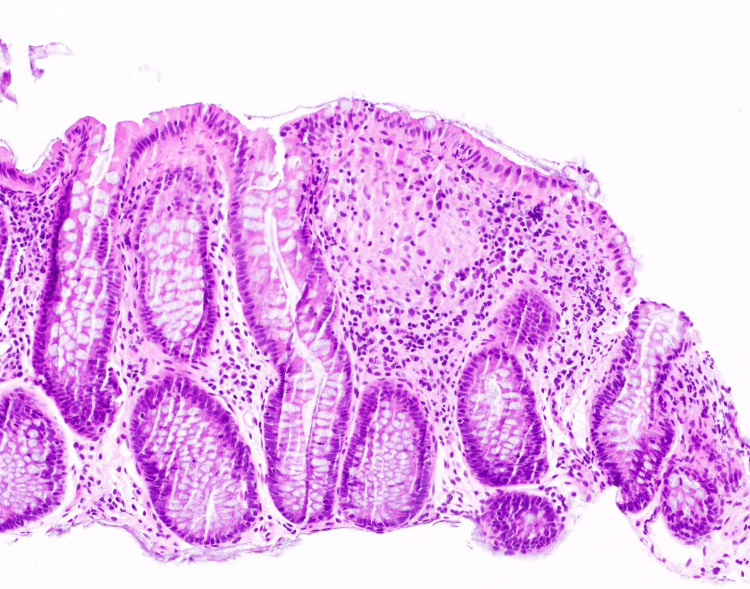
Colonic mucosa with a nonnecrotizing granuloma suggestive of CD CD: Crohn's disease

A colonoscopy showed terminal ileitis with ulcerations. A biopsy showed CD (Figure 4).

**Figure 5:**
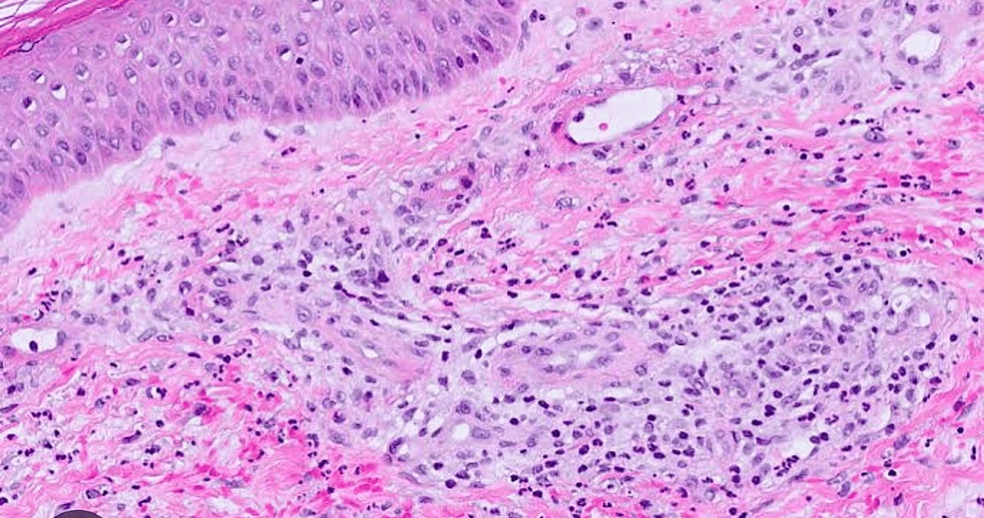
Skin with dermis showing perivascular neutrophilic infiltrate, fibrinoid necrosis, and extravasation of RBCs suggestive of leukocytoclastic vasculitis

After meticulous evaluation and considering the opinion of a gastroenterologist, it was decided to manage appendicitis conservatively. The case was taken over by rheumatology for further management of coexisting skin lesions and past history of arthritis. Skin biopsy showed leukocytoclastic vasculitis with IgA (Figure 5) [7].

The patient was managed with Inj. Dexa 8 mg and IV fluids. The patient improved symptomatically and was discharged with the Tab. Wysolone. The patient was doing well on follow-up.

## Discussion

The decision regarding the management of appendicitis should not be made in haste without a complete work-up of the patient. Age, coexisting symptoms, and history must be carefully evaluated before starting the treatment. In doing so, unnecessary surgery and hospital stays of the patient can be avoided. Though not clearly understood, the appendix plays a role in intestinal homeostasis, halting the development of certain disease pathologies [8]. Studies have shown that appendicectomy is positively associated with the development of CD, especially in the first years of surgery. Many studies have also noted that patients with CD with a history of appendicectomy had a worse prognosis and higher rate of intestinal resections compared to nonappendectomized patients [9]. Based on the endoscopic and CT findings, the European Crohn’s and Colitis Organisation has a set of guidelines that states that if there is no obstruction, granulomatous appendicitis can be handled like a localized ileocecal CD with medical management, which includes remission induction via systemic steroids [10].

## Conclusions

The presentation of Crohn’s with appendicitis is unusual and rarely seen. Correlating clinical and radiographic findings in diagnosing Crohn’s appendicitis is important. As seen in the above case, it is essential to consider all the symptoms at play in the patient before deciding on the line of management for appendicitis. The skin lesions and musculoskeletal symptoms should not be ignored, as doing so may prevent identifying the etiology of appendicitis. It is a well-known fact that CD is associated with skip lesions and therefore multiple biopsies should be taken for a positive histopathological diagnosis. The mainstay of management of Crohn’s is the use of steroids and it is evident in the present case as the patient improved symptomatically with the same.
